# Advancements in the Cultivation, Active Components, and Pharmacological Activities of *Taxus mairei*

**DOI:** 10.3390/molecules29051128

**Published:** 2024-03-02

**Authors:** Xinyu Gao, Ni Zhang, Weidong Xie

**Affiliations:** 1 State Key Laboratory of Chemical Oncogenomics, Shenzhen International Graduate School, Tsinghua University, Shenzhen 518055, China; gao-xy23@mails.tsinghua.edu.cn (X.G.); zhangn22@mails.tsinghua.edu.cn (N.Z.); 2 Shenzhen Key Laboratory of Health Science and Technology, Institute of Biopharmaceutical and Health, Shenzhen International Graduate School, Tsinghua University, Shenzhen 518055, China

**Keywords:** *T. mairei*, phytochemical constituents, pharmacological activity

## Abstract

*Taxus mairei* (Lemée and H.Lév.) S.Y.Hu, indigenous to the southern regions of China, is an evergreen tree belonging to the genus Taxus of the Taxaceae family. Owing to its content of various bioactive compounds, it exhibits multiple pharmacological activities and has been widely applied in clinical medicine. This article comprehensively discusses the current state of cultivation, chemical constituents, applications in the pharmaceutical field, and the challenges faced by *T. mairei.* The paper begins by detailing the ecological distribution of *T. mairei*, aiming to provide an in-depth understanding of its origin and cultivation overview. In terms of chemical composition, the article thoroughly summarizes the extracts and monomeric components of *T. mairei*, unveiling their pharmacological activities and elucidating the mechanisms of action based on the latest scientific research, as well as their potential as lead compounds in new drug development. The article also addresses the challenges in the *T. mairei* research, such as the difficulties in extracting and synthesizing active components and the need for sustainable utilization strategies. In summary, *T. mairei* is a rare species important for biodiversity conservation and demonstrates significant research and application potential in drug development and disease treatment.

## 1. Introduction

*Taxus mairei* (Lemée and H.Lév.) S.Y.Hu, *T. mairei*, a distinctive under-forest medicinal plant resource native to China, amalgamates medicinal, ornamental, and timber values. With a 2.5 million-year history on Earth, this species is globally recognized as an endangered and precious plant, often referred to as “plant gold” [1,2]. Originating from China, it is distributed across provinces such as Anhui, Zhejiang, Taiwan, Fujian, Jiangxi, Guangdong, Guangxi, Hubei, and Hunan, with presence also in India, Myanmar, Malaysia, Indonesia, and the Philippines [3]. As an evergreen tree unique to the southern regions of China, it has attracted widespread attention in the fields of pharmacology and biology in recent years. This rare plant plays a significant role in biodiversity conservation and holds immense application value in modern medicine due to its rich bioactive compounds. This review aims to comprehensively explore the research progress on *T. mairei*, from its cultivation status to chemical components, pharmacological activities, challenges faced, and future directions. It provides a thorough reference for researchers and professionals in related fields.

Firstly, this article will delve into the origin and cultivation conditions of *T. mairei*. Understanding its ecological distribution and environmental adaptability is critical to grasping biological characteristics and conservation value. A detailed description of its cultivation status reveals its position in the natural world and highlights the challenges in resource protection and sustainable utilization.

The phytochemical components of *T. mairei* will be deeply analyzed from three aspects: the chemical component characteristics of different parts of *T. mairei*, the impact of origin and growth duration on active components, and comparisons with other *Taxus* species in terms of chemical components. These studies not only aid in a deeper understanding of the chemical properties of *T. mairei* but also provide a significant scientific basis for developing its medicinal value.

This paper focuses on the pharmacological activity research of *T. mairei*. The potential applications of *T. mairei* in clinical medicine are revealed by analyzing the pharmacological effects of its extracts and monomeric components. Applying *T. mairei* in the medical field is also a research hotspot. Various bioactive substances are contained in different parts of it. Based on the latest scientific achievements, this section not only elucidates the mechanisms of action of *T. mairei* but also showcases its prospects as a new drug development candidate.

As a plant resource with significant medicinal value, the research on *T. mairei* is fundamental to the scientific community. It has profound implications for practical clinical applications and drug development. Future research should focus on its sustainable utilization and protection to ensure this precious resource can be appropriately conserved and utilized while serving human health. Through scientific research and technological innovation, the study of *T. mairei* will continue to bring breakthroughs and contributions to drug discovery and biodiversity conservation.

## 2. Origin and Cultivation

**Figure 1:**
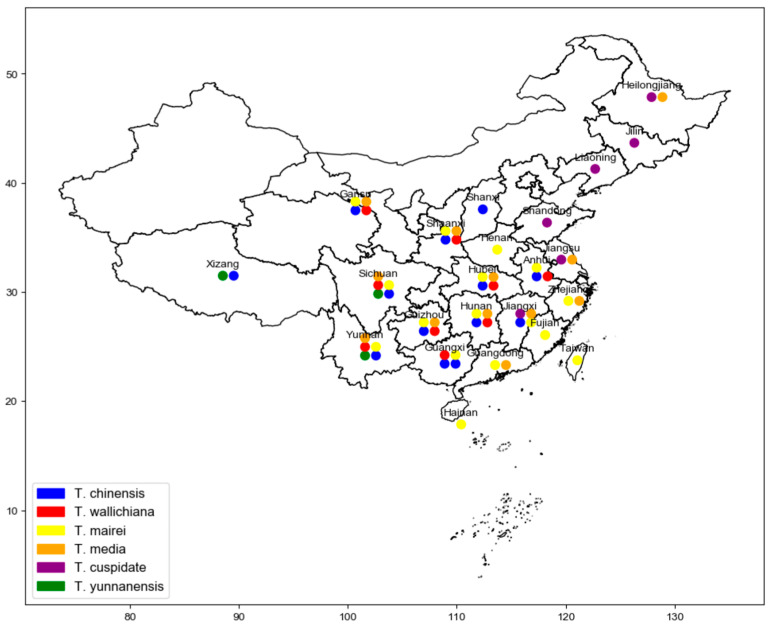
The geographical distribution of native and hybrid species of Taxus in China.

Taxus, belonging to the Taxaceae family, is one of the world’s precious tree species. Due to its unique ecological and medical values in China, Taxus has been classified as a first-class protected plant [4,5,6]. Globally, there are eleven species of Taxus, with China being home to four species and one variety, namely *Taxus chinensis* (Pilger.) Rehd, *Taxus mairei* (Lemée and H.Lév.) S.Y.Hu, *Taxus wallichiana* Zucc., *Taxus cuspidata* Siebold and Zucc., and *Taxus yunnanensis* W.C.Cheng and L.K.Fu. In addition, there are also the hybrid species *Taxus* × *media* Rehder [7,8]. Figure 1 shows the geographical distribution of native and hybrid species of Taxus in China.

*T. mairei* has an extensive distribution in China. It is primarily concentrated in Shaanxi, Gansu, and the areas south of the Yangtze River. *T. mairei* is found from the northern part of Guangdong and Guangxi to the southeastern part of Shanxi at altitudes of 600 to 1200 m in subtropical mountainous areas [9]. This species typically grows in mountains or valleys at 800 to 1600 m. The uniqueness of its growing environment makes *T. mairei* the most widely distributed Taxus species in China [10]. Studies have shown that this species has a solid adaptability to soil types but prefers well-drained, fertile soil conditions [11].

As a relict plant from the Tertiary period, *T. mairei* is excellently protected and propagated in the Nanling region of China. Along the Nanling mountain range, from the Yuanbao and Mao’er mountains in the north of Guangxi, through Hunan’s Dupang and Mang mountains, eastward to the Dayuling at the junction of Jiangxi and Guangdong, and to the southern end of Wuyi Mountain in western Fujian, natural populations of *T. mairei* can be found in this continuous geographical area [12]. Nature reserves play a significant role in biodiversity conservation, and there are nature reserves for *T. mairei* in various provinces in China, such as the Guangdong Ruyuan *T. mairei* Nature Reserve located in the subtropical climate zone and the Sichuan Tangjiahe National Nature Reserve [13]. Hubei province has wild *T. mairei* resources, but the growth rate could be faster and meet clinical needs. Therefore, artificial cultivation bases have been established in Enshi, Zhongxiang, and Xianning of Hubei province [14]. These bases provide valuable plant resources for the medical industry and play an essential role in protecting this rare species.

## 3. Phytochemical Components

### 3.1. Chemical Composition of Different Parts of T. mairei

*T. mairei* contains a wide variety of chemical components, with over 100 compounds identified from different parts of the plant to date, primarily consisting of taxanes, diterpenes, flavonoids, lignans, steroids, glycosides, phenolic acids, fatty acids, fatty alcohols, and polysaccharide compounds, among others [15]. Below, the chemical constituents of the leaves and twigs, bark, and seeds of *T. mairei* from various regions in China are discussed:

#### 3.1.1. Chemical Components of Leaves and Twigs of *T. mairei*

The leaves and twigs of *T. mairei* contain many chemical components, including taxanes, polysaccharides, amino acids, terpenes, and phenylpropanoids. The taxane compounds in the leaves and twigs are diverse. In addition to the representative component paclitaxel [16], the major taxane components in the leaves and twigs include 10-Deacetylbaccatin III, Baccatin III, Cephalomannine [17], Baccatin IV, 5-Decinnamoyltaxinine J, 2-Deacetoxytaxinine J, Adzukirin J, Taxinine B, 7-epi-10-deacetyltaxol, 1β-Hydroxybaccatin I, Taxayuntin F, Taxuspines A, Taxin B, Taxuspine W, Taxuspine B, among others [18,19]. The content of taxanes in the leaves and twigs is relatively high, with paclitaxel content ranging from 0.1136 to 0.1530 mg/g [20,21,22]. The content of 10-Deacetylbaccatin III in the leaves and twigs is the highest among other parts, with an average content of 0.0115% [23]. Baccatin III and 7-epi-10-deacetyltaxol contain 0.0156% [24] and 0.02372% [25]. The content of Cephalomannine is 0.7932 mg/g [20]. Baccatin III and Cephalomannine are higher in the leaves and twigs, serving as precursor substances for the synthesis of paclitaxel, which can be extracted from the leaves and twigs and obtained through semi-synthesis.

Besides taxanes, the leaves and twigs contain a wide variety of flavonoid compounds, mainly including Quercetin, Kaempferol, Isorhamnetin, Sciadopitysin, Myricetin, Ginkgetin, Amentoflavone, Sotetsuflavone, Kayaflavone, etc. The total flavonoid content measured within these medicinal materials can reach 9.31–36.46 µg/g [26]. The extraction amounts of Amentoflavone, Quercetin, and Ginkgetin from these medicinal materials are 4.47 mg/g, 3.73 mg/g, and 3.47 mg/g, respectively [27]. Although these plant materials may have potential applications in traditional medicine, considering the toxic compounds they contain, their use must be approached with extreme caution.

The leaves and twigs of *T. mairei* contain polysaccharides. The polysaccharide content varies from 4.52 to 45 mg/g depending on the extraction method [28]. For instance, the crude polysaccharides extracted from the leaves and twigs of *T. mairei* using the water-extraction alcohol-precipitation method showed a total sugar content of 30.51% measured by the sulfuric acid–phenol method, and the protein content measured by the Coomassie Brilliant Blue method was 4.52% [29]. The polysaccharide composition is glucose:mannose:xylose:arabinose:rhamnose: galactose (molar ratio of 1:0.32:0.27:3.34:1.22:1.84) [30]. The polysaccharide content in the leaves is higher than that in the twigs [28].

The leaves and twigs of *T. mairei* contain many volatile components, which differ between genders. The main components in male leaves are Z-3-hexen-1-ol (25.93%), (E)-2-hexenal (13.52%), and 1-Octen-3-one (12.71%), while in female leaves, the main components are (Z)-3-Hexen-1-ol (15.70%), 4-Hydroxybenzaldehyde (5.29%), and Octadecane (11.20%) [31]. The accumulation of paclitaxel in branches and leaves of female *T. mairei* (with red aril, FR) is significantly higher than that in males (M); however, its content decreases with age [32].

Biogenic volatile organic compounds (BVOCs) are natural volatile organic compounds released by plants [33]. BVOCs include many compounds, such as terpenes, phenols, and organic sulfur compounds. These compounds are crucial in plant growth and development and benefit human health [34]. The chemical components released by living leaves and twigs of *T. mairei* could identify 145 kinds of BVOCs, accounting for 96.29% of the total volatiles, with terpenes being the dominant relative content at 67.13% and alkanes accounting for 11% [35].

In the needles of *T. mairei*, taxane substances are also abundant, mainly including paclitaxel, 10-Deacetylbaccatin III, Baccatine III, 7-Xyl-10-DAT, 10-Deacetyltaxol, Cephalomannine, and 7-epi-10-deacetyltaxol, etc. [19,36]. Among them, the content of 10-Deacetylbaccatin III, 7-Xyl-10-DAT, and 10-Deacetyltaxol is relatively high, with studies determining their respective content to be approximately 626 µg/g, 546 µg/g, and 236 µg/g [37]. However, the content of these compounds may vary significantly depending on biological age, collection time, and other differing quantities of secondary metabolites. The average content of Baccatin is 391 µg/g, paclitaxel is 223 µg/g, and Cephalomannine is 107 µg/g [38]. The distribution of paclitaxel content in the needles of different varieties of *T. mairei* also varies; for example, in ‘Jinxishan’ (a cultivar from *T. mairei* with yellow aril, FY), the paclitaxel content in needles is relatively high but less in FY branches [32].

The needles of *T. mairei* also contain a certain amount of flavonoid components, with an average total flavonoid content of 59.327 ± 0.036 mg/g, including Taxifolin, Amentoflavone, Quercetin, and Ginkgetin, among others, with the maximum concentration of Taxifolin being 2540 µg/g [6]; the contents of Quercetin and Amentoflavone are close, with average contents of 0.047 mg/g and 0.040 ± 0.001 mg/g, respectively [38].

Fajun, C. reported that the total polysaccharide content in fresh needles of *T. mairei* is about 3.93% [39]. Fang, C. found that the average content in fresh needles is 20.864 ± 0.087 mg/g, which varies with the season [38]. The molecular weight of the polysaccharides is distributed within the range of 27.515 kDa, mainly composed of galacturonic acid, unknown sugar, rhamnose, arabinose, and glucose in the ratio of 2.18:13.98:6.85:8.64 [29].

#### 3.1.2. Chemical Components of the Bark of *T. mairei*

The bark of *T. mairei* contains a vast array of diterpenoid taxane compounds, including paclitaxel, Cephalomannine, 10-Deacetylbaccatin III(10-DAB), Taxusin, Decinnamoyltaxagifine, 19-Debenzoyl-19-acetyltaxinine M, 9-Dihydro-13-acetylbaccatin III, Baccatin III, Taxinine E, 7,9-Dideacetylbaccatin IV, 1,3-Dihydrotaxinine, Taxuyunnanine C, Taxuspine J, 7-xylosyl-10-deacetyltaxol A, 10-Deacetyltaxol, taxicin II, 2α,7β,10β-triacetoxy-5α,13α-dihydroxy-2(3→20)Abietaxa-4(20), and 11-dien-9-one, among others [40,41,42,43]. The total content of taxane compounds in the bark of *T. mairei* is 0.396 mg/g [21]. Research has found that the sequence of paclitaxel content in different parts of the yew from highest to lowest is bark, root bark, lateral branches, seeds, fibrous roots, young branches, and leaves, with the highest content found in the bark, averaging 418 µg/g [44]. The average content of 10-DAB in the bark of *T. mairei* is 0.0680% [45], the content of 7-xyl-10-DAT is 288 µg/g [46], and the content of Cephalomannine is 0.034 mg/g [43].

Additionally, the bark of *T. mairei* contains numerous trace elements, including K, Ca, Mg, Cu, Zn, Mn, Fe, Na, Cr, etc., with the highest contents being K, Ca, and Mg at concentrations of 1939.3, 1936.9, and 1409 µg/g, respectively [47].

#### 3.1.3. Chemical Components of the Fruit and Seeds of *T. mairei*

*T. mairei*, as a gymnosperm, does not possess actual flowers or fruits in the traditional sense. Its so-called “fruit” is constituted by a fleshy part surrounding the seed, distinctly different from the fruits of angiosperms. Traditionally, the focus has been more on the components of the leaves and twigs of *T. mairei* rather than its seeds or fleshy parts. However, with further research, these parts have garnered attention for containing various valuable chemical components.

The seeds of *T. mairei* are a vital medicinal resource of the plant, boasting a rich chemical composition [48]. The seed parts mainly contain soluble sugars, proteins, starch, crude fat, and amino acids. The content of soluble sugars is approximately 10.66%, soluble proteins about 0.68%, starch around 0.42%, crude fat about 19.74%, and amino acids about 9.1% [49,50]. The fatty acid composition in the seed oil is predominantly unsaturated oleic and linoleic acids, with their relative contents being 48.4% and 42.2%, respectively [51]. The seeds also contain taxane compounds, including paclitaxel, 7-epi-10-deacetyltaxol, 10-DAB, Taxinine A, Taxuspine X, Decinnamoyltaxinine E, 9-Deacetyltaxinine, 9-Deacetyltaxinine E, 2-Deacetyltaxinine, and 5alpha-cinnamoyloxy-9alpha,10beta,13alpha-triacetoxy-taxa-4, among others. Notably, paclitaxel and 7–epi–10–Deacetyltaxol have the highest contents in the seed embryo at 866.47 and 722.50 µg/g, respectively [25,52,53,54]. The contents of 10-Deacetylbaccatin III, Baccatin III, and Cephalomannine in the seed embryo are 124.09 µg/g, 44.88 µg/g, and 25.16 µg/g, respectively [25]. In the seed coat, the highest content of taxane compounds is paclitaxel, reaching up to 173.94 µg/g, with the contents of 10-Deacetylbaccatin III and Baccatin III being close at 116.05 and 116.60 µg/g [25,55], respectively.

The seeds of *T. mairei* are also rich in volatile components. The volatile components vary with different colors, with the main components in the green part being Z-3-hexen-1-ol (16.09%), 1-octen-3-ol (57.56%), and hexanol (6.17), and the red part being Heptadecane (8.47%), Octadecane (15.57%), and Nonadecane (9.12%) [31]. The elemental mass fraction in the seeds includes Nitrogen, Phosphorus, Potassium, Calcium, and Magnesium with contents of 87.2 mg, 565.4 mg, 261.1 mg, 18.4 mg, and 350.5 mg (per 100 g dry weight), respectively, with other trace elements from highest to lowest being Zinc, Iron, Sodium, Copper, Manganese [51].

Compared to the harm caused to plants by collecting bark and roots, using seeds or the fleshy part as a resource for Taxus medicine is more sustainable.

### 3.2. Factors Influencing the Active Components of T. mairei

Several factors influence the content of active components in *T. mairei*, including the plant’s origin, growth environment, age, harvesting season, and variety [56].

Active components vary among *T. mairei* from different origins. Peilei Bai et al. used HPLC to analyze the content of paclitaxel in leaves and twigs of twelve wild *T. mairei* samples from six origins, finding significant differences in paclitaxel content among samples from different origins. Samples from Shaanxi and Gansu had significantly higher paclitaxel content than those from Anhui, with the highest content in samples from Zhouzhi, Shaanxi (0.0193 mg/g). This difference is speculated to be related to environmental variations [57]. The content of taxane components in 3-year-old seedlings of *T. mairei* grown in greenhouses in Hunan and Zhejiang provinces was measured, finding that the contents of 10-DAB and Docetaxel (DXT) were higher, with Hunan’s 10-DAB and DXT contents being 1.65 mg/g and 1.5 mg/g, respectively, and Zhejiang’s being 0.75 mg/g and 1.45 mg/g, respectively, with other taxane component contents being similar [58].

The practical component content varies with different *T. mairei* growth years. Study results showed that the highest contents of paclitaxel and 10-DAB were found in three-year-old medicinal materials, being 0.4589 mg/g and 0.2920 mg/g, respectively. Among different harvesting periods of three-year-old *T. mairei* medicinal materials, the highest contents of practical components were found in June, with paclitaxel and 10-DAB contents being 0.5253 mg/g and 0.3972 mg/g, respectively [59].

The content of taxane compounds in Taxus varies with the harvesting period. A study on the variation pattern of paclitaxel content in the leaves and twigs of *T. mairei* showed significant changes from July to November, with paclitaxel content gradually increasing in July and August, respectively, at 0.383‰ (dry weight) and 1.219‰ (dry weight), and gradually decreasing from August to November, with paclitaxel contents being 1.219‰ (dry weight), 0.683‰ (dry weight), 0.591‰ (dry weight), and 0.209‰ (dry weight), reaching its peak in August at 1.219‰ (dry weight), which is 5.8 times the lowest value in November [60]. Zhaohui Wang et al. found that the highest paclitaxel content in *T. mairei* from Hunan was in June [61].

Different varieties of *T. mairei*, such as the different colors of the fleshy part surrounding its seeds, show differences in paclitaxel content in the bark. The research found that the average paclitaxel content in yew bark was 0.000564 mg/g, while in the bark surrounding the red fleshy part, the paclitaxel content was 0.000785 mg/g. In the bark surrounding the golden fleshy part, the paclitaxel content was 0.000703 mg/g [62].

### 3.3. Comparison of Chemical Components between T. mairei and Other Taxus Species

The taxane compound content in *Taxus* species, as shown in Table 1, highlights significant variations that have implications for their ecological adaptations and pharmaceutical value. For instance, *T. yunnanensis* exhibits the highest range of paclitaxel content, suggesting a strong potential for medicinal use. Conversely, the varied content of 10-DAB, Baccatin III, and Cephalomannine across species underscores the genetic diversity within the genus, affecting their selection for drug synthesis. This diversity not only reflects the adaptability and evolutionary history of *Taxus* species but also indicates their specific conservation needs and potential pharmacological applications.

## 4. Pharmacological Activity Studies

*T. mairei* has been listed as a provincial standard medicinal material in many provinces such as Zhejiang, Shanghai, Guangdong, Hunan, Jiangsu, etc. The “Zhejiang province Traditional Chinese Medicine Pieces Processing Specification” records the medicinal parts, taste, meridian tropism, and primary functions of *T. mairei*: the main medicinal parts are the branches with leaves, “slightly sweet, bitter, neutral, entering the kidney and heart meridians”, with the effects of reducing swelling, dispersing masses, promoting menstruation, diuresis, and clinically used for masses, edema, difficulty in urination, rheumatic pain, etc. [70]. Below, the anticancer, anti-inflammatory, antihypertensive, antidiabetic, and antimicrobial effects are described respectively:

### 4.1. Anticancer Activity

#### 4.1.1. Anticancer Activity of Extracts

*T. mairei* contains many active chemical components; thus, its total extract shows specific anticancer activity. The aqueous extract of *T. mairei* (AETC) has shown significant effects in both in vitro and in vivo studies in anticancer research.

In in vivo studies, AETC exhibited significant inhibition of NCI-N87 human gastric cancer cell xenograft tumors in nude mice and induced apoptosis. Administered via gavage at doses of 2.080–0.520 g/kg, AETC effectively inhibited the growth of NCI-N87 gastric cancer xenograft tumors in nude mice expressing high levels of HER2 and enhanced its inhibitory effect when combined with Herceptin treatment [71]. The immunoglobulin protein CD47 is overexpressed in malignant tumor cells, allowing them to evade host immunity by inhibiting macrophage-mediated phagocytosis. AETC reduced CD47 levels in non-small cell lung cancer (NSCLC) cells and Lewis tumor xenograft mice, enhancing immunity to NSCLC by triggering ubiquitination and degradation of CD47 [72]. In studies exploring the effect of AETC on the growth of A549 lung cancer xenografts in nude mice and its mechanisms, oral administration of AETC for seven weeks, compared to the control group, showed significantly reduced levels of EGFR and Survivin mRNA in the xenograft tissues, indicating a potential mechanism by which AETC inhibits tumor growth by affecting these molecular targets [73]. Research on the effect of the aqueous extract of *T. mairei* combined with Erlotinib on the growth of A549 tumor xenografts in nude mice found that, compared to the control group, the tumor weight in the experimental group significantly decreased, with marked reductions in EGFR mRNA expression, COX-2 mRNA expression, Bcl-2 mRNA expression, and COX-2 protein expression [74].

In in vitro studies, AETC and paclitaxel exhibited significant inhibitory effects on gastric cancer cells SGC-7901 and breast cancer cells MCF-7, showing a dose–effect relationship. Studies indicated that the IC50 of AETC for these two cell types were (2.23 ± 0.13) mg/mL and (2.29 ± 0.15) mg/mL, respectively, demonstrating its significant inhibitory effect on tumor cell proliferation [75]. Further research showed that AETC inhibited NSCLC cell proliferation, especially significant in lung cancer cells NCI-1975, and could induce apoptosis. The anti-tumor effect of AETC is associated with the upregulation and downregulation of ATF3 expression, involving inhibiting the Hippo pathway and reducing YAP degradation. AETC also reduced tumor volume and weight in nude mice, upregulated ATF3, p-MOB1, and p-YAP (Ser397), actively regulated cleaved PARP, and caspase-9/8/3, showing its role in inducing apoptosis in NSCLC cells in vitro and in vivo through the ATF3-Hippo-YAP pathway [76]. The MTT assay detected the inhibitory effect of *T. mairei* Aqueous Extract (TAE) and paclitaxel on the proliferation of gastric cancer cells SGC-7901 and breast cancer MCF-7 cells. The effect of TAE on the morphology of SGC-7901 and MCF-7 cells was observed under a microscope. The results indicated that the extract of *T. mairei* inhibits tumor cell proliferation, which is related to inducing tumor cell apoptosis [75]. Additionally, the CCK-8 assay showed that TAE has a selective inhibitory effect on the growth of non-small cell lung cancer A549 and HCC827 cells and is dose-dependent, with minimal impact on everyday human lung cells. Its mechanism may be related to inhibiting tumor cell proliferation and metastasis by inactivating the JAK/STAT3 axis [77,78]. Research also indicated that taxane compounds extracted from *T. mairei* exhibit a specific inhibitory effect on the proliferation of A549 non-small cell lung cancer cells (IC50 between 26–167 μg/mL). Additionally, these compounds showed potent inhibitory activity against B16 mouse melanoma cells (IC50 between 20–768 μg/mL) and a strong inhibitory effect on the proliferation of BEL7402 human hepatoma cells (IC50 between 30–273 μg/mL) [79].

#### 4.1.2. Anticancer Activity of Monomers and Major Effective Components

*T. mairei* is rich in taxane compounds with anticancer effects [80]. Paclitaxel, as the most critical anticancer monomer in the taxane series of *T. mairei*, has been widely used in the treatment of various cancers since its approval by the FDA in December 1992, becoming a recognized effective broad-spectrum anticancer drug [81,82,83]. The IC50 of paclitaxel against tumor cells in vitro ranges from 2.5 to 7.5 nM [84]. Paclitaxel inhibits cancer cell division and proliferation by stabilizing microtubule structure and preventing the normal function of microtubules during mitosis. Additionally, paclitaxel can block the cell cycle in the G2/M phase, further inhibiting cancer cell growth [85,86].

In clinical applications, postoperative treatment of breast cancer patients with paclitaxel can effectively reduce recurrence and mortality rates, further confirming the significant effect of paclitaxel in breast cancer treatment [87]. In ovarian cancer treatment, paclitaxel is used as a second-line drug in dose-dense regimens for salvage therapy after relapse [88]. Approximately 80–85% of lung cancers are pathologically classified as non-small cell lung cancer (NSCLC). Paclitaxel, by interfering with microdynamics, is a first-line chemotherapeutic drug for treating advanced NSCLC [89]. These study results fully demonstrate the multifaceted role and clinical value of paclitaxel in treating different cancers.

Other *T. mairei* components, such as 10-DAB, Cephalomannine, etc., also possess good anticancer activity. 10-DAB is a crucial precursor compound in *T. mairei*, providing an essential intermediate for synthesizing paclitaxel-like drugs. Studies indicate that while 10-DAB has weak anticancer activity, it is indispensable in developing potent anticancer drugs such as paclitaxel and docetaxel as a precursor [90]. Cephalomannine, an alkaloid with anticancer activity, has an IC50 of 1.458–1.499 µg/mL [91,92] and is a derivative of the taxane diterpene class, mainly reported in *Taxus* species [92]. Research has explored the effect of Cephalomannine on lung cancer cells under hypoxic conditions, finding it inhibits lung cancer cell growth, reactive oxygen species (ROS) production, intracellular pH, and migration, as well as angiogenesis of HUVECs under hypoxic conditions by inhibiting the APEX1/HIF-1α interaction [91]. Additionally, Taxinine from *T. mairei* also has anticancer activity [93], with IC50 values against tumor cells A549, B16, and BEL7402 being 46.17, 350.64, and 113.97 µg/mL, respectively [79,94]. Studies have shown that Baccatin III has anti-tumor immunomodulatory activity at very low doses (0.05–0.5 mg/kg). Oral Baccatin III significantly reduced tumor growth induced by 4 T1 breast cancer or CT26 colon cancer cell transplantation in BALB/c mice by reducing tumor progression by inhibiting the accumulation and inhibition of MDSCs [95]. However, the monomer activity of the above components is weaker than paclitaxel, and they have not been developed into marketed anticancer drugs alone, mainly serving as intermediates for the synthesis of paclitaxel.

With more profound research into *T. mairei*, it has been found that taxanes are not the only components with anti-tumor activity; some polysaccharides, and among various compounds, flavonoids were specifically noted for their ability to inhibit cancer cell proliferation. These flavonoids show dose-dependent antiproliferative activities, effectively inducing apoptosis in cancer cells. Studies demonstrated that at concentrations ranging from 55.51 to 82.75 µg/mL, these flavonoids significantly inhibit the growth of human breast cancer MDA-MB-231 cells, underscoring the critical importance of dosage in their anti-tumor efficacy [96,97]. Taxus polysaccharides can inhibit S180 sarcoma, HepA liver cancer, and Lewis lung cancer, significantly improve mice’s hypoxia tolerance, enhance endurance, and increase survival rate [98]. Cultured human cervical cancer HeLa cells in the logarithmic growth phase were treated with different concentrations of Taxus polysaccharides (30,60,90,120 μg/L), resulting in a significant increase in cell proliferation inhibition rate and apoptosis rate compared to the control group, possibly related to downregulating Survivin, Bcl–2, and Caspase–3 expression and upregulating P53 expression [99]. Zhao’s study indicated that polysaccharides extracted from the fruits of *T. mairei* showed a 76.33% inhibition rate against S180 cells, with no toxicity to organs such as the liver, kidney, and heart [49]. The polysaccharide component PSY-1 can inhibit tumor growth in mouse models of S180 sarcoma, Lewis lung cancer, and HepA liver cancer, potentially related to inhibiting the expression of matrix metalloproteinases MMP-2 and MMP-9 and the phosphorylation of Iκβ [100]. Total flavonoids in *T. mairei* can enhance the inhibitory effect of paclitaxel on mouse breast cancer 4T1 and lung cancer A549 cells. Total polysaccharides can enhance the inhibitory effect of paclitaxel on breast cancer MCF-7 cells and mitigate the myelosuppressive effect of paclitaxel, with the most significant inhibitory effect on S180 sarcoma activity at a dose of 0.4 mL 66.6 mg/mL total polysaccharides, 0.4 mL 20 mg/mL total flavonoids, and 0.1 mL 1.25 mg/mL paclitaxel, with an inhibition rate of 38.86% [101]. Related research studies suggest that while *T. mairei* polysaccharides exert potent anti-tumor effects, their impact on non-cancerous, benign cells appears minimal, indicating a selective toxicity profile [102,103]. This selectivity is paramount for therapeutic agents to ensure efficacy against cancer cells while preserving the integrity and function of healthy tissues.

The *T. mairei* extracts, mainly when used in conjunction with other drugs, also demonstrate excellent anticancer effects. This is particularly the case with paclitaxel. The combination of paclitaxel and cisplatin shows significant effects against various cancers, and the combined chemotherapy of paclitaxel and carboplatin is a first-line cancer chemotherapy regimen for ovarian cancer. Liu and others treated 40 cases of ovarian cancer with a combination of paclitaxel and cisplatin, supplemented by comprehensive nursing interventions, achieving a total effective rate of 95% [104]. Another study involving 110 ovarian cancer patients treated with a combination of paclitaxel and cisplatin chemotherapy found significant therapeutic effects, with a total of 76 compelling cases, accounting for 98.70% [105]. In studies involving 40 lung cancer patients each, intravenous drip of 135 mg/m^2^ paclitaxel and 70 mg/m^2^ cisplatin on days 7 and 14 showed that the combination treatment had a significant effect on lung cancer, with higher rates of gastrointestinal reactions, leukopenia, thrombocytopenia, and bone marrow suppression in the treatment group compared to the control group [106]. The combination of paclitaxel and emodin has a synergistic inhibitory effect on the proliferation of A549 cells in vitro. Increasing the expression of Bax and active caspase three and reducing Bcl-2, p-Akt, and p-ERK levels significantly promotes ptx-induced apoptosis in A549 cells [107]. Research by Li and others found that the aqueous extract of *T. mairei* combined with paclitaxel can also inhibit the growth of human lung cancer A549 cells, downregulate the expression of Bcl-2 and Survivin genes, and upregulate Bax expression [108]. The aqueous extract of *T. mairei* used in combination with erlotinib enhances the effect of erlotinib, possibly through the downregulation of COX-2 and MMP-2 protein expression [109].

### 4.2. Antidiabetic and Antihypertensive Effects

Recent studies have revealed the significant potential of extracts from the leaves and twigs of *T. mairei* in regulating blood sugar, protecting organs, and their antioxidant properties. Research indicates that alcoholic extract and crude polysaccharides from *T. mairei* leaves and twigs can effectively inhibit weight loss, significantly reduce fasting blood glucose levels, regulate dyslipidemia, and protect the liver, kidney, and pancreas in diabetic rats. These components also improve glucose tolerance, demonstrating their potential for diabetes management. Different extract fractions have varied effects on blood sugar reduction in normal and insulin-resistant HepG2 cells, highlighting the importance of concentration in their efficacy. Further studies found that different extract fractions from the leaves and twigs of *T. mairei* have varying effects on reducing blood sugar. In normal HepG2 cells, the alcoholic extract and crude polysaccharides showed the best antidiabetic effects at 0.05 mg/mL. At the same time, the ethyl acetate and n-butanol fractions were most effective at a concentration of 0.01 mg/mL. For the HepG2 cell insulin resistance model, the n-butanol fraction and crude polysaccharides were most effective at reducing blood sugar at a concentration of 0.05 mg/mL, with ethyl acetate and alcoholic extracts being most effective at a concentration of 0.01 mg/mL [29].

Studies on the fruits of *T. mairei* also showed good antioxidant and anti-hyperglycemic activities and the potential for safety and bioactive components. The antioxidant activity of *T. mairei* fruit is good, with a DPPH radical scavenging rate of 82.1%, slightly lower than that of Vitamin C (96.04%) but still showing significant effects; its hydroxyl radical scavenging ability is lower than Vitamin C, with an EC50 of 1.306 mg/mL. Acute oral toxicity tests in mice indicated that the methanol extract of yew is safe [48].

Yang W X’s team extracted volatile components from fresh *T. mairei* leaves and tested them on a rat model of hypertension. By administering the extract orally at a dose of 5 mg/kg once daily for six weeks, it was found that the treatment significantly inhibited the increase in systolic blood pressure and plasma angiotensin II in rats. Although it did not significantly reduce blood triglycerides (TG), high-density lipoprotein cholesterol (HDL-C), and low-density lipoprotein cholesterol (LDL-C), it reduced total cholesterol (TC) and dose-dependently increased plasma NO levels [110].

### 4.3. Anti-Inflammatory Effects

*T. mairei* exhibits significant anti-inflammatory activity, notably due to its terpenoid content, which is recognized for its potent anti-inflammatory properties. These terpenoids function by inhibiting various upstream kinase signaling pathways (such as TLR, RAGE, TNFR, and IL-6R receptors), MAPK/p38/ERK/JAK signaling, HMGB-1 release, NF-κB activation, translocation, or reducing NF-κB DNA binding ability, thereby suppressing inflammation [111]. The NF-κB signaling pathway is crucial in regulating the expression of a wide array of cytokines involved in airway inflammation, remodeling, asthma, and other respiratory diseases [112].

In studies targeting specific components, the ethyl acetate fraction of the Southern Yew fruit showed a definite anti-inflammatory effect. Administering 200 mg/kg/day of this extract to twenty female SD rats for four weeks resulted in a significant decrease in the serum levels of IL-1β, IL-6, and IL-18 compared to the control group [113]. One of the polysaccharides from the Southern Yew (PTM) has been found to inhibit oxidative stress and apoptosis. Treating C57BL/6J mice modeled with Alzheimer’s disease (AD) with PTM (0.4 g/kg/day) for 14 days resulted in reduced levels of MDA and ROS, increased expression of NRF2, and improved cognitive functions, thus suggesting that PTM could reduce neurotoxicity and cognitive dysfunction [114]. The ethyl acetate extract of the Southern Yew fruit significantly lowered the serum levels of IL-1β, IL-6, and IL-18 after administration to SD rats at 200 mg/kg/day, demonstrating its anti-inflammatory effect [114]. The stems and leaves of the Southern Yew have certain analgesic and anti-inflammatory effects, with a clinical dose of 8 g/(case·day), equivalent to 0.13 g/kg/day (assuming a human body weight of 60 kg). Oral administration to Kunming mice at doses ranging from 0.133–0.667 g/kg/day resulted in inhibition of pain induced by thermal stimuli, with analgesic rates of 23.77–47.83%, and a reduction in the number of writhes induced by acetic acid in mice [115]. The carrageenan-induced rat paw edema model, a standard model for screening anti-inflammatory drugs, was used with an ethanol-percolated extract from the Southern Yew stems and leaves rich in terpenoids. This was prepared into a topical application for SD rats and ICR mice models. The study found that the Southern Yew ethanol extract inhibited paw swelling in rats at all tested doses, with the high dose group showing significant suppression of paw swelling, superior to the positive control drug, suggesting its potential development into a topical formulation for treating local swelling or arthritis [116].

Regarding the effects of individual components, one of the polysaccharides from the Southern Yew (PTM) used in treating C57BL/6J mice modeled with Alzheimer’s disease (AD) was found to lower MDA and ROS levels, increase NRF2 expression, and improve cognitive functions, showing its effectiveness in inhibiting oxidative stress and apoptosis [110]. Incubation with different extracts from the Southern Yew, including taxane and volatile oil components, could regulate the NF-κB signaling pathway, thus playing a role in inhibiting airway inflammation [117]. Taxifolin is a bioflavonoid which has been used to treat Inflammatory Bowel Disease. Taxifolin prevented the increase in serum aminotransferase activity during inflammation [118]. Treatment of mice with Taxifolin and fecal transplantation showed a lower diarrhea score, reduced colonic inflammation, and less mucosal damage, possibly related to increased levels of butyrate in fecal metabolites [119]. Amentoflavone, a biflavonoid naturally occurring compound, exhibits significant anti-inflammatory properties. It has demonstrated efficacy in mitigating pilocarpine-induced epileptic seizures in mouse kindling models by inhibiting nuclear factor-κB (NF-κB) activation and expression. This inhibition curtails the excessive firing of hippocampal neurons, thereby reducing the frequency and duration of epileptic episodes. Additionally, amentoflavone contributes to decreasing neuronal loss and apoptosis in the hippocampus, further underscoring its potential therapeutic benefits in epilepsy management [120]. Baccatin III, an essential precursor to paclitaxel, exhibits notable anti-inflammatory effects with reduced toxicity. The findings revealed that Baccatin III administration, in a dose-dependent manner, lessened inflammatory infiltration and the release of the pro-fibrotic mediator TGF-β1. It also decreased the accumulation of collagen and various extracellular matrix (ECM) components, such as alpha smooth muscle actin (α-SMA) and fibronectin [121].

### 4.4. Antimicrobial Effects

Extracts from *T. mairei*, specifically flavonoid-rich fractions, have demonstrated antimicrobial activities. Fifty-nine flavonoid compounds have been identified from *T. mairei*, demonstrating significant pharmacological activity [122]. A study exploring the antifungal effects of amentoflavone showed vigorous antifungal activity against several pathogenic fungi but low hemolytic activity against human red blood cells. As a stress response to the drug, amentoflavone induced trehalose accumulation inside Candida albicans cells and disrupted pseudohyphae formation during pathogenesis, showing potential as a lead compound for antifungal drug development [123]. Additionally, amentoflavone’s impact on inducing mitochondrial dysfunction and apoptotic cell death in Candida albicans has been investigated [124]. Ginkgetin, another flavonoid from these extracts, has been noted for its anticancer properties, including cell cycle arrest, apoptosis induction, autophagy stimulation, and interference with dysregulated pathways such as JAK/STAT and MAPKs [125]. Moreover, Quercetin 3-O-β-D-glucoside has been identified to inhibit HIV-RT activity with an IC50 value of 50 μmol/L, showcasing anti-HIV virus effects [126].

In our previous discussion, we focused on the efficacy of components found in *T. mairei*, such as anticancer, anti-inflammatory, and antihypertensive effects. However, the cytotoxic compounds present in the Taxus genus, including various alkaloids, especially taxine, possess certain toxicity to the human body, particularly affecting cardiac function, which may lead to decreased blood pressure and bradycardia. Studies have indicated that the leaves and seeds of *T. mairei* are toxic, and excessive intake could lead to symptoms ranging from dizziness and abdominal pain to severe life-threatening conditions such as cardiac arrest. The lethal oral dose of yew leaves in humans is 0.6–1.3 g per kg, equivalent to 3.0–6.5 mg of taxine per kg [127]. Even small amounts of extract from the Taxus genus can result in severe consequences. Taxine A and taxine B, two primary alkaloids found in the Taxus genus [128], are rapidly absorbed by the human body and primarily affect cardiac function, causing a decrease in blood pressure and, in large amounts, leading to serious toxic reactions such as cardiac arrest and respiratory distress [127]. Therefore, while the active effects of plant extracts are worth attention, we must also consider their potential toxicity in application. Comprehensive toxicity assessment in the development and use of plant extracts is crucial. Understanding the mechanisms of action and risks of these toxic components is essential for preventing accidental poisoning and ensuring their safe and effective medical use. Future research should include systematic toxicity assessments to better understand the safe application range of these extracts, thereby maximizing their pharmacological activity while ensuring patient safety.

## 5. Discussion and Conclusions

**Figure 2:**
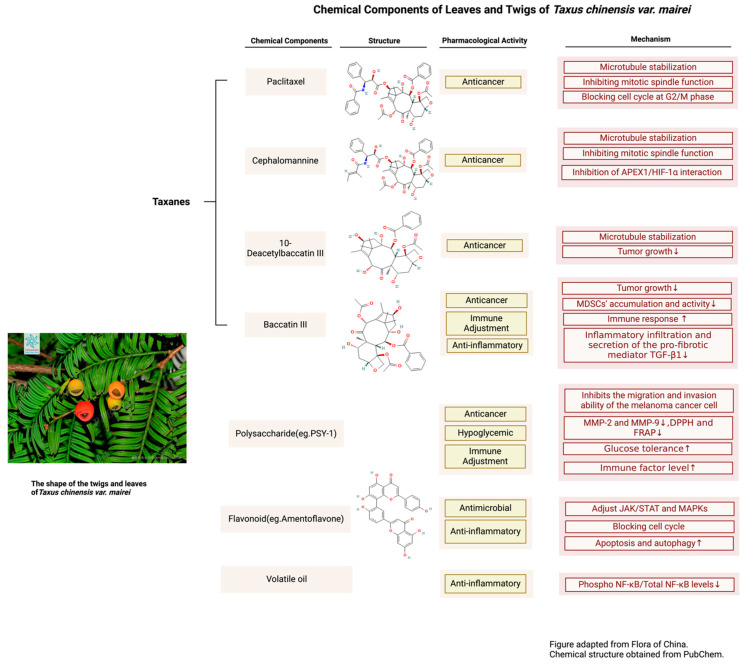
Chemical Components of Leaves and Twigs of *T. mairei*.

This article comprehensively discusses the current status, challenges, and prospects of *T. mairei* in botany, chemistry, and pharmacology. As a rare medicinal plant, *T. mairei* plays a crucial role in biodiversity conservation and exhibits significant potential in medical research due to its unique chemical components. Through an integrated analysis of the growth environment, chemical diversity, and pharmacological effects of *T. mairei*, this study highlights the urgency of conducting in-depth research and conservation of *T. mairei*, as well as its importance in new drug development (Figure 2).

In phytochemistry, this article analyzes the progress in research on *T. mairei*, revealing the diversity of its medicinal resources, the complexity of its plant secondary metabolites, and the uniqueness of their biosynthesis. Paclitaxel and its derivatives are recognized anticancer drugs as the main active components in *T. mairei*. Moreover, *T. mairei* contains other various alkaloids and compounds, such as Cephalomannine, 10-DAB, flavonoids, and polysaccharides, which are distributed differently across the plant parts, offering the potential for more efficient drug extraction based on these differences. However, apart from paclitaxel, these components’ pharmacological effects and interactions have yet to be fully revealed. Current research tends to focus on the overall study of extracts from *T. mairei* rather than specific chemical components, leading to relatively general and insufficiently deep investigations. Future research should also delve deeper into these secondary metabolites’ biosynthetic pathways, molecular structures, and interactions to unveil their potential pharmacological value. Presently, the extraction and separation techniques for active components of *T. mairei* need improvement to enhance the efficiency and purity of component extraction.

Current research has predominantly focused on exploring the effects of extracts from *T. mairei* without a detailed investigation into the specific chemical components within these extracts. This approach has led to a somewhat generalized and superficial understanding of the plant’s potential medicinal benefits. As we move forward, it is imperative to shift our focus towards a more granular examination of the individual compounds present in the extracts. This refined focus will not only allow us to identify the bioactive components contributing to the extracts’ therapeutic effects but also to isolate these beneficial compounds while eliminating those with toxic properties. Such a shift would enable us to decipher the complex interactions between these compounds and their collective impact on pharmacological efficacy. Future research should strive to identify and characterize the full spectrum of bioactive compounds in *T. mairei*, delineating their molecular structures, biosynthetic pathways, and pharmacological actions. Actually, the action mechanisms of most extracts and even well-defined compounds remain unclear and require further investigation in the future. This comprehensive understanding is crucial for optimizing the medicinal use of *T. mairei*, enhancing drug development processes, and ensuring the safety and efficacy of derived pharmaceuticals.

To ensure the quality and consistency of extracts, *T. mairei* extracts must undergo strict quality control. Quality control refers to the process of ensuring consistency in the biological activity and chemical composition of each batch of extract by determining the concentration of one or more key active components. Using standard methods to prepare extracts often involves advanced analytical techniques such as High-Performance Liquid Chromatography (HPLC) to accurately quantify the content of specific components in the extract. It also establishes methods for controlling the content of bioactive components, such as paclitaxel and 10-Deacetylbaccatin III, using thin-layer chromatography for identification, among others [127]. Bioactive components like paclitaxel are often targeted for quality control, with their accurate quantification being crucial for assessing the medicinal value and safety of the extracts. With these measures, the efficacy and safety of different batches of extracts can be ensured, thereby minimizing potential risks due to fluctuations in ingredient concentrations. By strictly controlling the content of active ingredients in the extract, we can greatly reduce the safety risks during the use of patients, ensuring that the application of the extract is both safe and effective. However, in previous studies, most of the pharmacological studies of extracts did not conduct a well-defined quality control to establish a reliable standardization of extract pharmaceutics and need further improvement in the future.

Further research should aim to develop more efficient and environmentally friendly extraction methods and explore new pathways to synthesize these complex compounds, reducing dependence on natural resources. Future studies should also focus on the effects of *T. mairei* extracts in different disease models and their interactions with human metabolism and physiological processes. Given the safety and efficacy of plant-derived drugs, future research also needs to include toxicological evaluation and clinical trials of these extracts.

Although significant achievements have been made in studying *Taxus* species’ chemical components and pharmacological properties, most research has focused on the compound paclitaxel. Research on taxane compounds and other active components could be more extensive. Moreover, the limitations in selecting subjects and research periods have restricted a comprehensive understanding of the trends in active components. Therefore, in-depth and systematic analysis of active substances such as taxane compounds, flavonoids, and polysaccharides in the needles of *T. mairei* is of profound significance for the comprehensive development and utilization of yew resources. In this context, conducting more comprehensive research becomes particularly important. Research should not only consider paclitaxel but also cover taxane compounds and other potential active components to reveal the overall pharmacological properties of yew.

Additionally, research expansion should include different types and growth stages of yew samples and long-term observations to ensure more comprehensive and dynamic data. Such research will provide more accurate information for drug development and help formulate effective yew conservation and sustainable utilization strategies. Future research directions should focus on improving cultivation techniques and quality control and enhancing extraction efficiency of medicinal components. Strengthening the protection and study of the *T. mairei* ecosystem is also crucial.

In conclusion, while significant strides have been made in understanding the chemical composition and pharmacological benefits of *T. mairei*, there remains a vast frontier of research to be explored. The focus on paclitaxel, although crucial, has overshadowed the potential of other bioactive compounds within the plant. A more granular examination of these compounds, their biosynthetic pathways, molecular structures, and pharmacological actions is essential for unlocking the full medicinal potential of this rare species. Moreover, enhancing extraction efficiency, improving quality control measures, and exploring new methods for compound synthesis are critical steps towards sustainable utilization and conservation of *T. mairei*. As we advance, incorporating comprehensive research approaches that include different yew types, growth stages, and long-term observations will provide a more detailed understanding, facilitating drug development, biodiversity conservation, and human health improvement. Strengthening the ecosystem’s protection and studying *T. mairei* in depth will pave the way for new discoveries in drug development and disease treatment, showcasing the plant’s invaluable contribution to both biodiversity and the medical field.

## Figures and Tables

**Table 1:** Comparison of Taxane Content in the Leaves and Twigs of Different *Taxus* Species.

Species	Paclitaxel Content (mg/g)	10-DAB Content (mg/g)	Baccatin III Content (mg/g)	Cephalomannine Content (mg/g)
*T. wallichiana*	0.10–0.30 [20,63]	0.71 [20]	0.19 [20]	0.18 [20]
*T.* *yunnanensis*	0.52–1.00 [20,64]	0.700–0.821 [20,65]	0.83 [20]	0.01–0.12 [20,66]
*T. mairei*	0.11–0.15 [20,21,22]	0.74 [20]	0.01–0.44 [20,21]	0.23 [20]
*T. cuspidata*	0.14–1.67 [20,21]	0.77 [20]	0.16–0.77 [20,21]	0.25–0.84 [20,66]
*T. media*	0.60–1.20 [20,58,64,67]	0.34–0.75 [20,58]	0.29–0.30 [20,67]	0.53–0.60 [20,58]
*T. chinensis*	0.60 [68]	0.70–1.20 [68,69]	0.21–0.35 [68,69]	0.40 [68]

## Data Availability

The data presented in this study are available in the article.
